# Inhibitory Effects of Intranasal Administration of Insulin on Fat Oxidation during Exercise Are Diminished in Young Overweight Individuals

**DOI:** 10.3390/jcm7100308

**Published:** 2018-09-28

**Authors:** Hisayo Yokoyama, Ryosuke Takeda, Eriko Kawai, Akemi Ota, Emiko Morita, Daiki Imai, Yuta Suzuki, Tomoaki Morioka, Masanori Emoto, Masaaki Inaba, Kazunobu Okazaki

**Affiliations:** 1 Research Center for Urban Health and Sports, Osaka City University, 3-3-138, Sugimoto, Sumiyoshi-ku, Osaka 558-8585, Japan; wind-05@med.osaka-cu.ac.jp (R.T.); dimai@sports.osaka-cu.ac.jp (D.I.); suzuki@sports.osaka-cu.ac.jp (Y.S.); okazaki@sports.osaka-cu.ac.jp (K.O.); 2 Environmental Physiology for Exercise, Osaka City University Graduate School of Medicine, 3-3-138, Sugimoto, Sumiyoshi-ku, Osaka 558-8585, Japan; kawai@respiratorycontrol.com (E.K.); e-morita@pt-u.aino.ac.jp (E.M.); 3 Department of Health and Sports Science, Osaka Electro-Communication University, Osaka 572-8530, Japan; ota@osakac.ac.jp; 4 Metabolism, Endocrinology and Molecular Medicine, Osaka City University Graduate School of Medicine, 1-4-3, Asahi-machi, Abeno-ku, Osaka 545-8585, Japan; m-tomo@med.osaka-cu.ac.jp (T.M.); memoto@med.osaka-cu.ac.jp (M.E.); m1356849@med.osaka-cu.ac.jp (M.I.)

**Keywords:** intranasal insulin administration, central nervous system, fat oxidation, graded exercise, overweight, lipolysis

## Abstract

It remains unknown whether the high insulin (INS) levels in the brain affect fat oxidation during exercise. We examined the effects of the intranasal administration of INS, which increases the INS concentration in the cerebrospinal fluid when peripheral effects are lacking, on the maximum fat oxidation rate (maxFOR) and its intensity (FATmax) during exercise in 15 young normal-weight (N group) and eight young overweight (O group) individuals. On two separate days, either INS or placebo (PL) was randomly administered intranasally before a graded exercise test. Indirect calorimetry was used to assess maxFOR and FATmax during exercise. Blood INS and glucose levels did not change after INS administration. In the N group, maxFOR and FATmax were significantly smaller in the INS trial than in the PL trial. MaxFOR was significantly smaller in the O group than in the N group and was not influenced by INS administration. Exercise-induced elevation in blood epinephrine levels tended to be reduced by INS administration only in the N group. Intranasal INS administration reduces fat oxidation during exercise without any peripheral effects, possibly by suppressing sympathetic nerve activity. This inhibitory effect is diminished in overweight subjects, suggesting that cerebral insulin effects are attenuated in this population.

## 1. Introduction

During endurance exercise, the skeletal muscle must use both fat and carbohydrates as energy sources. Which of the substrates is predominantly used depends mainly on the exercise intensity [1,2]. In graded exercise, fat oxidation increases according to increasing exercise intensity, and then comes to a peak (maxFOR) when exercise reaches a specific (generally low) intensity, after which it conversely decreases and is gradually replaced by carbohydrate metabolism; therefore, exercise at the intensity that induces maxFOR can benefit people who are striving to lose body fat [3]. However, maxFOR and the exercise intensity at which maxFOR occurs are lower in obese individuals in relation to their insulin (INS) resistance compared with those in subjects of normal weight [4,5]. This “low metabolic fitness” might explain, at least in part, an impaired exercise capacity or the reduced effect of aerobic exercise on reducing body fat in obese subjects.

We know that INS in the central nervous system (CNS), as well as peripherally plays a role in the regulation of energy homeostasis. Peripheral INS secreted by pancreatic β-cells not only acts on the receptors distributed throughout skeletal muscles, liver, and adipose tissue, but also reaches CNS across the blood–brain barrier (BBB) by both receptor-mediated saturable transport [6] and receptor-independent manner [7]. INS receptors are also expressed in human CNS [8], and are primarily located in the cerebral cortex, olfactory bulb, hippocampus, cerebellum, and hypothalamus [9]. Animal studies have shown that INS infusion into the cerebral ventricle reduces blood glucose levels and food intake [10,11].

Substances that are intranasally administered are directly delivered to CNS through uptake into olfactory sensory neurons followed by axonal transport or extracellular transport along the olfactory nerves without substantial absorption into the blood stream [12]. In fact, the intranasal administration of INS also increases INS levels in the cerebrospinal fluid (CSF) without any relevant peripheral effects in humans [13,14], if it is administered at the dose of 40 units or lower [15]. Thus, the administration of intranasal INS has enabled a noninvasive examination of the role of CNS-specific INS functions in humans. Notably, the intranasal administration of INS acutely reduces the appetite and food intake [16] and chronically reduces the body fat in healthy subjects [17]. These results suggest that INS in CNS contributes to the regulation of eating behavior and energy metabolism, especially to facilitating catabolic action, as opposed to the effects of peripheral INS; however, it is unclear whether INS delivery to CNS influences fat oxidation during exercise.

Considering the above background information, we hypothesized that the intranasal administration of INS promotes fat oxidation during subsequent exercise in humans, even in cases of deteriorated peripheral insulin sensitivity; therefore, the objective of the present study was to investigate the impact of the intranasal administration of INS on fat oxidation in association with changes in energy sources and hormones during a graded exercise test in healthy young normal-weight and overweight subjects.

## 2. Materials and Methods

### 2.1. Subjects

Twenty-four healthy young adults between the ages of 18 and 40 were recruited from among the students in Osaka City University, Japan. Excluded were subjects who had a history of ischemic heart disease, chronic heart failure, hypertension (resting systolic blood pressure (SBP) ≥ 140 mmHg and/or diastolic BP (DBP) > 90 mmHg), diabetes, or nasal disorders. The body mass index (BMI) of the participants ranged from 16.4 to 32.3 kg/m^2^. The underweight (BMI < 18.5 kg/m^2^, *n* = 4) and normal-weight (18.5 ≤ BMI < 25.0 kg/m^2^, *n* = 11) subjects were assigned to the normal-weight (N) group (BMI < 25.0 kg/m^2^, *n* = 15, 7 males and 8 females), and the others to the overweight (O) group (BMI ≥ 25.0 kg/m^2^, *n* = 8, 4 males and 4 females). Age, percent body fat, and peak oxygen uptake (VO_2_) were not different between the underweight and the normal-weight subjects except for BMI (17.5 ± 0.8 and 19.8 ± 1.1, respectively. *p* = 0.002). Written informed consent was obtained from all the participants. The study protocol was approved by the Institutional Review Board of Osaka City University Graduate School of Medicine (approval No. 3205) and was registered at the University Hospital Medical Information Network-Clinical Trial Registry (study ID: UMIN000018941). This study also conformed to the ethical principles regarding human experimentation developed by the Declaration of Helsinki.

### 2.2. Study Design

**Figure 1:**
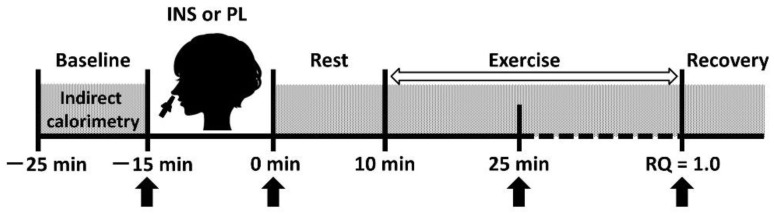
Experimental protocol. Hatched square indicates indirect calorimetry. Blood samplings are indicated by black arrows. Notes: INS, insulin; PL, placebo; and RQ, respiratory quotient.

Our investigation was a randomized, single-blind, crossover study. All the subjects visited our laboratory at Osaka City University on 2 separate days at least 1 week apart but within a period of 2 weeks for the two trials (i.e., the INS and placebo (PL) trials) in random order. Figure 1 shows the study protocol.

All the experimental sessions were conducted in the TBR-6W2S2L2M climatic chamber (ESPEC Co., Osaka, Japan) at a constant temperature of ~25 °C and a relative humidity of ~40%. In each trial, the experimental session began at 9:00 am after a 12-h fast. First, respiratory gas was analyzed as a baseline measurement. Then, INS or PL was administered intranasally. Analysis of the respiratory gas continued throughout the graded exercise. An intravenous cannula was placed in the cubital vein and blood samples were collected for the baseline measurements levels of glucose, INS, free fatty acid (FFA), glycerol, epinephrine, norepinephrine, and leptin. Blood sample evaluations of these levels were repeated immediately after intranasal dosing, 15 min after beginning the graded exercise (except for leptin), and at the termination of the graded exercise. SBP and DBP, as well as heart rate (HR) were also recorded at 1-min intervals throughout the exercises.

### 2.3. Determination of Peak VO_2_

At least 1 week before the first trial, all the subjects underwent maximum-ramp incremental tests to exhaustion on the AEROBIKE^®^75XLIII cycle-ergometer (Konami Sports Life Co., Ltd., Zama, Kanagawa, Japan) to determine the peak VO_2_. At least 3 h after the last meal, the subjects exercised on the cycle-ergometer after a 3-min rest in the sitting position. The initial exercise load was fixed at 20 watts for a 3-min warm-up stage and was subsequently increased by 20 watts per min. Pedaling was maintained at 60 rpm throughout the duration of the test. Expiratory gas was measured breath by breath to evaluate VO_2_ (mL/min) and carbon dioxide production (VCO_2_, mL/min) using the AE-310S electronic spirometry system integrated with a gas analyzer (Minato Medical Science, Osaka, Japan). SBP/DBP and HR were continuously monitored throughout the test using the EBP-330 automated sphygmomanometer (Minato Medical Science, Osaka, Japan) and the Life scope 8 BSM7106 electrocardiograph (Nihonkoden, Osaka, Japan), respectively. The rating of the perceived exertion (RPE) using the Borg scale was evaluated each minute. We considered the peak load to have been attained when the subjects met at least two of the following criteria [18]: (1) HR of 85% of the age-predicted maximum, (2) RPE of ≥18, (3) respiratory quotient (RQ, VCO_2_/VO_2_) ≥ 1.1, and (4) no further VO_2_ increase regardless of load increase. The average VO_2_ in the last 30 s of the graded test was defined as the peak VO_2_.

### 2.4. Intranasal Administration

After the analysis of the baseline respiratory gas and blood sampling, four 10-unit puffs (alternating two per naris; total dose: 40 units INS) of Novolin^®^ R regular INS (Novo Nordisk Pharma, Bagsvӕrd, Denmark) or 0.1 mL normal saline (total dose: 0.4 mL) for the PL trials, were administered intranasally in 1-min intervals. INS and PL were administered using a plastic nasal pump spray bottle (AS ONE Co., Osaka, Japan), which filled the nasal cavity with aerosol, enabling the solution to effectively target the olfactory epithelium. The administered dose of the intranasal INS used here has been shown to be effective in stimulating INS receptors in CNS without affecting the peripheral blood INS levels in healthy humans [15].

### 2.5. Respiratory Gas Analysis during the Graded Exercise Test to Assess Fat Oxidation Rate

Respiratory gas was analyzed using the AE-310S electronic spirometry system integrated with a gas analyzer (Minato Medical Science Co., Osaka, Japan) to evaluate VO_2_, VCO_2_, and RQ at 1-min intervals. After a 5-min rest on the cycle-ergometer in a sitting position, VO_2_ and VCO_2_ were assessed for 10 min. Baseline VO_2_, VCO_2_, and RQ were determined by averaging those parameters in the last 5 min of this 10-min baseline period. Fifteen minutes after the first puff of INS or PL, respiratory gas analysis was restarted. The VO_2_, VCO_2_, and RQ at rest were also determined by averaging their values during the 10-min rest period. FOR at 1-min intervals during the exercise test was estimated using the following formula by Frayn [19]:

FOR (mg/kg/min) = 1.67 × VO_2_ (mL/min) − 1.67 × VCO_2_ (mL/min) (1)

Graded exercise was continued until RQ reached 1.0, the point at which it was believed that fat oxidation was terminated and that carbohydrates were used as the predominant fuel source.

SBP, DBP, and HR at the baseline and at rest were determined using the same method used with the respiratory parameters. SBP, DBP, and HR levels 15 min after beginning the graded exercise were determined by averaging their values in 11–15 min of the exercise period.

### 2.6. Estimation of maxFOR and FATmax

**Figure 2:**
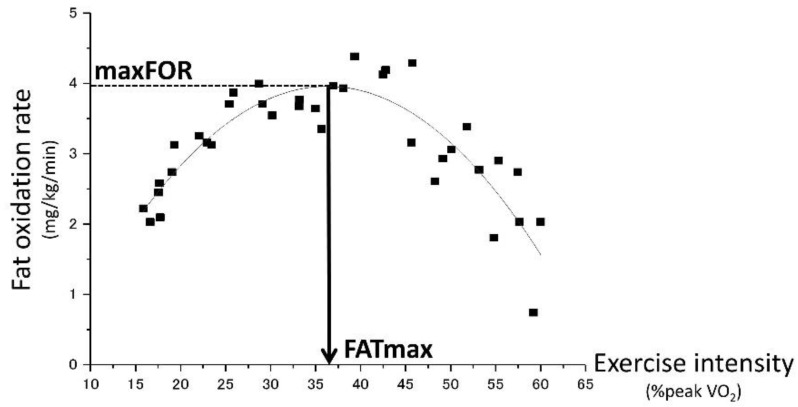
Estimating the maximum fat oxidation rate (maxFOR) and FATmax. For each subject, a quadratic polynomial curve was constructed by plotting all 1-min records on a graph with FOR (mg/kg/min) versus exercise intensity, which is represented by % peak oxygen uptake (VO_2_). Curve fitting and peak analysis were conducted using software to determine the maxFOR during the incremental exercise test and FATmax, the exercise intensity that raises the maxFOR. Notes: FOR, fat oxidation rate; and VO_2_, oxygen consumption.

As Figure 2 shows, maxFOR and FATmax were estimated from the results of FOR and the concurrent VO_2_ during the incremental exercise test. For each subject, a quadratic polynomial curve was constructed by plotting each 1-min record on a graph with FOR (mg/kg/min) versus exercise intensity, which is represented by %peak VO_2_. ORIGIN^®^2017 (LightStone, Tokyo, Japan) was used for curve fitting and peak analysis to determine the maxFOR during the incremental exercise test and FATmax, the exercise intensity at which maxFOR occurs.

### 2.7. Anthropometrical Measurements

Percent body fat was estimated by bioelectrical impedance analysis using the TBF-102 Body Fat Analyzer (TANITA, Tokyo, Japan) before the first experimental session. BMI was calculated as body weight (kg) divided by height (m) squared.

### 2.8. Laboratory Measurements

The collected blood samples were centrifuged for 15 min at 3000 rpm, and the resulting serum and plasma were stored at −80 °C until assay. The hexokinase ultraviolet method was used to measure plasma glucose levels. Chemiluminescent enzyme immunoassay was used to measure the serum INS levels, and the enzymatic (acyl-CoA synthase and ketoamine oxidase, respectively) methods were used to measure the FFA and glycated albumin levels. High-performance liquid chromatography was used to measure the plasma epinephrine and norepinephrine levels. Serum glycerol levels were measured using the Glycerol Colorimetric Assay Kit (Cayman Chemical Company, Ann Arbor, MI, USA) according to standard protocols. Regarding the performance of the kit, both intra- and interassay coefficients of variation (CVs) were <10%. The plasma leptin levels were also measured using the Human Leptin (highly sensitive) Assay Kit (Immuno-Biological Laboratories, Gunma, Japan). The intra- and interassay CVs were <15% and <20%, respectively. We calculated the homeostasis model assessment of insulin resistance (HOMA-IR), an established surrogate index [20], from fasting blood samples in the PL trial. HOMA-IR was obtained from fasting plasma glucose (FPG) and fasting serum INS (FIRI) levels according to the original method developed by Matthews et al. [21] using the following formula:

HOMA-IR = FPG (mmol/L) × FIRI (μU/mL)/22.5(2)

A higher HOMA-IR value represents higher insulin resistance.

### 2.9. Statistical Analyses

The data are presented as the mean ± standard error of mean (SEM) unless otherwise indicated. The effects of the trial (administration of intranasal PL or INS) and above-normal weight on the parameters for fat oxidation during graded exercise were examined by two-way (trial × group) analysis of variance (ANOVA) with repeated measurements; this procedure was repeated for both the PL and the INS trials. The effects of the graded exercise, trial, and above-normal weight on blood parameters were examined by three-way (time × trial × group) ANOVA with repeated measurements. This procedure was repeated for both successive intervals within and between the PL and INS trials. Multiple post-hoc pairwise comparisons (Dunnett’s test) were conducted if there was a significant time effect. If there was a significant trial effects, subsequent comparisons were conducted using a paired *t*-test. An unpaired *t*-test was used for comparisons between the groups. SPSS v 24 (IBM Corp., Armonk, NY, USA) for Windows (Microsoft Inc., Redmond, WA, USA) was used to perform all the statistical procedures. *p* values < 0.05 were considered statistically significant.

## 3. Results

Among the 25 applicants, 24 who met the inclusion criteria were enrolled in the study. One female applicant who showed impaired fasting glucose (6.7 mmol/L) was excluded from the study. Table 1 summarizes the clinical characteristics of all the subjects. Fifteen subjects were assigned to the N group, and eight were assigned to the O group. Body weight, BMI, and percent body fat in the O group were significantly greater than those in the N group. None of the other clinical parameters were different between the two groups.

As Table 2 shows, BP and HR during the graded exercise were not affected by the intranasal administration of INS in either group (all *p* > 0.05 for time × trial interaction). RQ at the baseline in the O group was significantly smaller than that in the N group (0.81 ± 0.04 (SD (standard deviation)) vs. 0.82 ± 0.05, respectively; *p* = 0.003). No effects of the intranasal administration of INS on VO_2_, VCO_2_, and RQ were observed in either group, except that RQ in the N group increased from 0.81 ± 0.04 at baseline to 0.84 ± 0.06 at rest after intranasal administration of INS (time × trial interaction *p* = 0.020).

**Figure 3:**
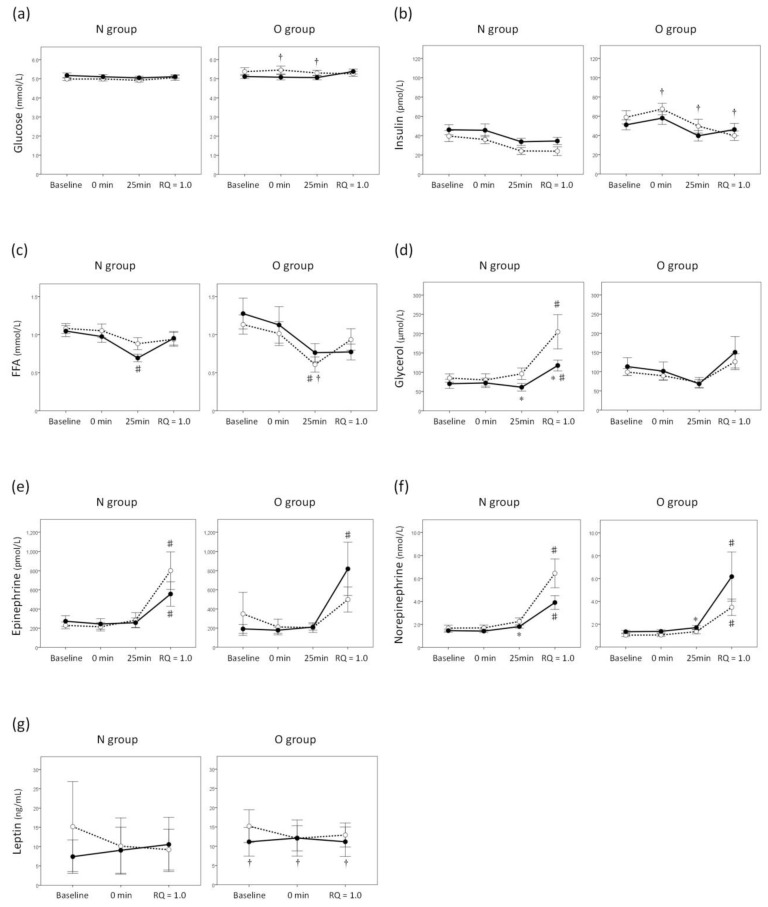
Changes in blood parameters during the graded exercise in both trials. Blood glucose (**a**), insulin (**b**), free fatty acid (FFA) (**c**), glycerol (**d**), epinephrine (**e**), norepinephrine (**f**), and leptin (**g**) levels in the placebo (PL) and the insulin (INS) trials are indicated by white and black circles, respectively. All values are presented as the means ± SEM. ^#^: *p* < 0.05 vs. baseline. *: *p* < 0.05 vs. PL. ^†^: *p* < 0.05 vs. N group.

Neither the blood glucose nor the insulin levels were affected by either exercise or the intranasal administration of INS in either group (Figure 3a,b). Table 3 shows the parameters for fat oxidation during graded exercise in both groups. MaxFOR in the O group was significantly smaller than that in the N group. MaxFOR and FATmax, as well as total exercise time and total amount of fat oxidation during the graded exercise were significantly smaller in the INS trial than in the PL trial in only the N group. The group × trial interactions with all these parameters were significant, suggesting that the effect of intranasal administration of INS on fat oxidation during graded exercise was different between the groups.

Blood levels of FFA in the INS trial in the N group and those in the PL trial in the O group were decreased after 25 min compared with the baseline (Figure 3c). Blood levels of glycerol in both trials in the N group were increased at the end of the graded exercise compared with the baseline (Figure 3d). In the INS trial, blood levels of glycerol after 25 min and at the end of the graded exercise in the N group were smaller than those in the O group (Figure 3d). However, significant time × trial interactions were found on neither FFA nor glycerol levels, which meant that the changes in blood FFA and glycerol levels during the graded exercise were not affected by intranasal administration of INS in either group. The effects of the intranasal administration of INS on blood epinephrine and norepinephrine levels during graded exercise were different between the groups (*p* = 0.017 and *p* = 0.020 for time × trial × group interactions on epinephrine and norepinephrine levels, respectively). In the N group, the increase in blood epinephrine levels during graded exercise tended to be reduced by intranasal administration of INS (*p* = 0.050 for time × trial interaction, Figure 3e). The main effect of the trial on the blood norepinephrine levels was also significant only in the N group (*p* = 0.025), and the levels in the INS trial after 25 min were smaller than those in the PL trial (Figure 3f). Blood leptin levels were not affected by either exercise or intranasal administration of INS in either group (Figure 3g).

## 4. Discussion

The aim of the present study was to examine the effects of a high INS state in CNS on fat oxidation during the graded exercise using the intranasal administration of INS, which enables the assessment of CNS-specific INS functions. Our main findings were that fat oxidation during exercise was reduced by the intranasal administration of INS without increasing peripheral insulin levels in our normal-weight healthy young subjects, and that this inhibitory effect was diminished in overweight subjects.

To the best of our knowledge, this is the first report to examine the effect of the intranasal administration of INS on fat oxidation during exercise. It was first reported in 2002 that the intranasal administration of INS allows direct access to the brain without affecting peripheral INS levels in humans [13]. Later, evidence regarding the role of INS in CNS has been accumulated using intranasal administration. Several studies have examined the effect of a single dose of intranasal INS on eating behavior and demonstrated a reduction in appetite as well as palatability [22], food intake [16,23], and food image-cued brain activity [24]. It was also shown that a single dose of intranasal INS increased postprandial diet-induced thermogenesis in healthy men [25]. Furthermore, an eight-week intranasal INS treatment resulted in the reduction of body weight and fat in healthy men [17]. Based on these previous findings which suggested the catabolic effect of INS in CNS, we expected that the intranasal administration of INS augments fat oxidation during exercise; however, contrary to our previous expectations, the intranasal administration of INS reduced fat oxidation during exercise in our normal-weight subjects, and this result was the same as that found in peripheral hyperinsulinemia [26]. INS in the peripheral blood is transported to CSF across BBB [6]; therefore, the level of INS in CSF peaks later than that in the peripheral blood after a meal [27]. Based on this phenomenon, it is possible that the INS in peripheral blood and CSF complimentarily modulate energy homeostasis at specific time intervals. Indeed, previous studies on diabetic subjects have shown that the intranasal administration of INS reduced postprandial hyperglycemia by up to four h [28,29]. Body weight and fat loss observed in a previous study after intranasal INS treatment might not have resulted from enhanced catabolism, but by its anorexic effects. Finally, INS in the brain appears to contribute to regulating fat metabolism in the same way as peripheral INS.

In the present study, increase in blood epinephrine levels during exercise tended to be reduced by intranasal administration of INS in the N group, although both BP and HR throughout the experiments were not different between the two trials; therefore, the suppression of sympathetic nerve activity could mediate, at least in part, the reduction in fat oxidation during exercise caused by INS in the brain. Catecholamines (epinephrine and norepinephrine), as well as INS, have been considered to play a primary role in regulating lipolysis in humans [30]. Exercise activates the sympathetic nervous system, which amplifies lipolytic responses by activating various lipases, such as hormone-sensitive lipase in adipocytes [31]; however, it has not been elucidated which catecholamine predominantly contributes to lipolysis during exercise. A previous study by de Glisezinski et al. [32] demonstrated that blocking β–adrenergic receptors in subcutaneous adipose tissue reduced exercise-induced lipolysis, and that the effect was not found after infusion with octreotide, which blocks exercise-induced epinephrine, but not norepinephrine, elevation in the blood. The researchers concluded that epinephrine, predominantly derived from the adrenal medulla, is the major contributor to lipolysis during exercise. Our results were consistent with theirs in that fat oxidation during exercise was reduced in the INS trial, which also impaired the elevation of exercise-induced epinephrine, but not norepinephrine, in the blood of our normal-weight subjects. On the other hand, in an animal study, INS infusion into the cerebral ventricle and hypothalamus under a pancreatic clamp reduced lipolysis represented by glycerol appearance, and the effect disappeared after surgical and pharmacological sympathectomy. The findings suggest that brain INS suppresses lipolysis by decreasing the activity of noradrenergic neurons that innervate white adipose tissue. It is possible that the method by which the sympathetic nervous system modulates lipolysis depends on the state of physical activity (i.e., rest or exercising), and that this might have played a role in the above discrepancy. Due to methodological limitations, exercise-induced lipolysis has generally been estimated in only subcutaneous white adipose tissue, and the possibility of controlling exercise-induced lipolysis of visceral fat by brain INS is unknown. In addition, further investigations are needed to clarify whether INS in the brain modulates lipid utilization in skeletal muscle cells, one of the determinants of fat oxidation during exercise. Animal studies demonstrated that vagal nerve stimulation augments not only lipolysis in adipose tissue, but β-oxidation by upregulating peroxisome proliferator–activated receptor α, which is followed by an increase in energy expenditure [33,34]. The effect of intranasal INS on lipid utilization including vagal modulation was not elucidated in the present study, although the blood levels of leptin, known to facilitate lipid utilization in skeletal muscle cells, were not affected by either exercise or intranasal INS.

Studies have investigated the effect of anthropometrical factors on substrate oxidation during exercise. MaxFOR and FATmax were lower in obese subjects [5,35], especially in those with increased abdominal fat mass [36], than in normal-weight subjects. This “low metabolic fitness” [36] is believed to decrease the outcome of exercise training in the population [37]. The results of the present study were consistent with those of previous studies by showing less reduced fat oxidation during exercise in subjects with high BMI. Most of these studies concluded that lipolysis in obese subjects was impaired based on the fact that the increase in their blood glycerol levels per body fat mass during exercise was reduced. The impaired lipolysis is mainly from hyperinsulinemia, although poor responses of blood epinephrine to exercise in obese subjects might also contribute to impaired lipolysis in cases of an extended duration of endurance exercise [38]. On the other hand, FFA availability appears to be intact because a large amount of fat mass could compensate for the impaired lipolysis in obese subjects [5]; therefore, reduced fat oxidation in obese subjects during exercise is explained, at least in part, by a reduced capacity for lipid utilization in skeletal muscle. Indeed, human studies have clarified that not only the mitochondrial content in skeletal muscle cells but the mitochondrial enzyme activities, which are responsible for the uptake and oxidation of fatty acid chains, are decreased in obese subjects or patients with type 2 diabetes [39].

Few reports have suggested the lack of response to intranasal administration of INS in obese subjects and type 2 diabetes. The intranasal administration of INS improves peripheral INS sensitivity [40] and reduces blood flow in the region of the brain that corresponds to the desire for sweet food [41] in lean men, while these effects are weakened in overweight or obese men. It was also reported that the lipid content in hepatocytes was decreased by intranasal administration of INS in lean healthy subjects, but not in patients with type 2 diabetes [42]. Our results support those findings by demonstrating that the inhibitory effects of intranasal administration of INS on fat oxidation during exercise are diminished in overweight subjects. Based on their findings, Tschritter et al. [43] suggested that CNS insulin resistance in overweight humans, which showed suppressed cortical activity that was assessed by magnetoencephalogram during hyperinsulinemic-euglycemic clamping in overweight subjects compared to that in lean subjects, is correlated to the amount of body fat and peripheral insulin resistance. On the other hand, peripheral hyperinsulinemia does not necessarily result in increased levels of INS in CNS. The transport of INS into the brain across BBB is decreased with obesity [44] and type 2 diabetes [45] most likely to protect the brain from certain substances that could modulate metabolism [46] and to maintain brain homeostasis. All these results confirm the existence of CNS insulin resistance by showing the decreased response to intranasal administration of INS, which directly increases the levels of INS in the brain in overweight or obese subjects or those with type 2 diabetes. In fact, diet-induced obese rats have decreased insulin receptor density in the hypothalamus and fail in anorexic responses to INS infusion into the cerebral ventricle [47]. Plasma INS levels and HOMA-IR in the O group were greater than those in the N group in the present study with no statistical significance. It could be possible that a certain correlation between peripheral insulin sensitivity and that in the brain is found in the case of obvious difference in insulin sensitivity between the groups due to the increased number of subjects. Of course, there remains a possibility that intranasal INS could not further reduce fat oxidation during graded exercise in the overweight subjects because their maxFOR and FATmax were initially lower compared to those in the normal-weight counterparts.

There were some limitations in the present study. First, the numbers of subjects in both of the groups were small and unbalanced due to the low prevalence of overweight and obesity in our university students. These might not have been enough to verify the effect of above-normal weight on how intranasal administration of INS modulates blood parameters during graded exercise. Second, some previous reports regarding the effect of intranasally administered insulin suggested the gender difference: Intrasnasal administration of insulin resulted in decreased food intake [23] and body weight loss [17] in men, but not in women. While no difference was found in gender ratio between the groups in the present study, individual assessments in each gender are needed in the future to clarify the effects of intranasal administration of INS on fat oxidation during exercise. Third, we could not measure the INS levels in CNS directly; therefore, it is possible that the 40 units INS caused the heterogeneous elevation of INS levels in CNS among the subjects, and the differences in the INS levels in CNS might have affected the results in the present study; however, we adopted the INS dose according to that used in a previous report, which verified the high INS levels in CSF using the same dose of INS as that administered in our study [13].

## 5. Conclusions

The intranasal administration of INS reduces fat oxidation during exercise without increasing peripheral insulin levels, possibly by suppressing sympathetic nerve activity and lipolysis in adipose tissue. The inhibitory effects of the intranasal administration of INS on fat oxidation during exercise are diminished in overweight subjects, suggesting that cerebral insulin effects are attenuated in this population. Further studies are needed to elucidate the role of INS in CNS as a regulator of fat utilization in skeletal muscle cells, which is one of the determinants of fat oxidation during exercise.

## Figures and Tables

**Table 1:** Clinical characteristics of the subjects.

		N Group			O Group			*p*
*n* (male/female)	---	15 (7/8)			8 (4/4)			0.879 ^§^
Age	(years)	20.6	±	3.7	23.6	±	7.1	0.191
BW	(kg)	53.8	±	8.8	74.5	±	7.6	<0.001
BMI	(kg/m^2^)	19.2	±	1.5	27.2	±	2.5	<0.001
Body fat	(%)	16.7	±	5.3	33.6	±	9.2	<0.001
Peak VO_2_	(ml/kg/min)	37.7	±	7.7	31.9	±	9.1	0.119
Glucose	(mmol/L)	5.1	±	0.4	5.4	±	0.6	0.162
Insulin	(pmol/L)	41.7	±	22.9	59.0	±	19.4	0.079
Glycated albumin	(%)	13.4	±	1.3	12.9	±	0.9	0.348
HOMA-IR	---	1.38	±	0.81	1.99	±	0.61	0.072

All values are presented as *n* or mean ± standard deviation (SD). ^§^: by chi-square test. Abbreviations: N, normal-weight; O, overweight; BW, body weight; BMI, body mass index; VO_2_, oxygen excretion; HOMA-IR, homeostasis model assessment of insulin resistance.

**Table 2:** Changes in blood pressure and heart rate by intranasal administration and during graded exercise.

		N Group						O Group					
		PL			INS			PL			INS		
		Baseline	Rest	25 min	Baseline	Rest	25 min	Baseline	Rest	25 min	Baseline	Rest	25 min
SBP	(mmHg)	111 ± 15	111 ± 15	118 ± 24	113 ± 13	109 ± 19	123 ± 19	116 ± 20	122 ± 8	129 ± 15	118 ± 17	117 ± 16	130 ± 18
DBP	(mmHg)	73 ± 10	75 ± 11	71 ± 17	74 ± 12	77 ± 11	70 ± 12	88 ± 16 ^†^	80 ± 16	83 ± 8	83 ± 15	83 ± 19	79 ± 16
HR	(beats/min)	74 ± 10	75 ± 12	102 ± 16	77 ± 13	79 ± 12	104 ± 17	74 ± 9	76 ± 9	96 ± 12	77 ± 13	77 ± 12	99 ± 11

All values are presented as n or mean ± SD. ^†^: *p* < 0.05 compared with the N group. Abbreviations: PL, placebo trial; INS, insulin trial; SBP, systolic blood pressure; DBP, diastolic blood pressure; HR, heart rate. Other abbreviations are as above.

**Table 3:** Parameter regarding fat oxidation during graded exercise in both trials.

		N Group						O Group						*p*		
		PL			INS			PL			INS			Group	Trial	Group × Trial Interaction
MaxFOR	(mg/kg/min)	3.72	±	0.30	3.00	±	0.20 *	2.44	±	0.19 ^†^	2.69	±	0.27	0.035	0.166	0.007
FATmax	(%peak VO_2_)	38.5	±	2.5	32.1	±	1.9 *	33.0	±	3.4	36.0	±	4.4	0.837	0.354	0.017
Total exercise time	(min)	35.5	±	3.9	28.1	±	3.2 *	34.0	±	4.4	38.3	±	5.8	0.463	0.447	0.009
Total amount of FO	(g)	6.9	±	1.5	4.4	±	0.9 *	5.8	±	1.3	7.4	±	2.0 **	0.626	0.451	0.004

All values are presented as mean ± standard error (SE). *: *p* < 0.05 compared with the PL trial. **: *p* = 0.050 compared with the PL trial. ^†^: *p* < 0.05 compared with the N group. Abbreviations: FOR, fat oxidation rate; FATmax, exercise intensity that raises max FOR; FO, fat oxidation. Other abbreviations are as above.
